# Carbon Dots Doped with Dysprosium: A Bimodal Nanoprobe for MRI and Fluorescence Imaging

**DOI:** 10.3390/jfb9020035

**Published:** 2018-05-18

**Authors:** Timur Sh. Atabaev, Zhonglie Piao, Anara Molkenova

**Affiliations:** 1 Department of Chemistry, School of Science and Technology, Nazarbayev University, Astana 010000, Kazakhstan; anara.molkenova@nu.edu.kz; 2 Wellman Center for Photomedicine, Harvard Medical School and Massachusetts General Hospital, Boston, MA 02114, USA; zpiao@mgh.harvard.edu

**Keywords:** carbon dots, dysprosium, bimodal nanoprobes, fluorescence, magnetic resonance imaging MRI

## Abstract

In recent years, functional nanoprobes with multiple imaging modalities have become an emerging field of biomedical research. In this preliminary study, we utilized a facile hydrothermal method for the preparation of magneto-fluorescent bimodal carbon dots doped with dysprosium (Dy-CDs). The prepared Dy-CDs have shown a good colloidal stability in a water solution and strong blue–green fluorescence, with a maximum at 452 nm. In addition, the excellent transverse relaxivity of the prepared Dy-CDs (r_2_ = 7.42 ± 0.07 mM^−1^s^−1^) makes them also suitable for T_2_-weighted magnetic resonance imaging (MRI). Thus, synthesized Dy-CDs could be potentially utilized for both MRI and fluorescence imaging of living cells.

## 1. Introduction

The development of new nanoscale contrast agents is a promising areas of research in the field of nanomedicine. In particular, these multifunctional nanoprobes can be more accurate than their molecular counterparts, because of the greatly improved image contrast. To date, various nanostructures, such as Fe_3_O_4_ [1,2], MnO [3] and various lanthanide oxides [4,5,6,7], have been successfully tested as new bimodal nanoprobes for magnetic resonance (MR) and fluorescence imaging of living cells. On the other hand, the toxicity concerns of these inorganic nanoprobes limit their wide usage in medical applications. To solve the toxicity issue, an appropriate surface coating should be employed with an organic layer [8], or with some inert materials, such as carbon [9] and silica [10]. However, surface modifications may affect the relaxivity properties of paramagnetic nanoprobes and, as a result, reduce the image contrast [10]. Therefore, one can use a different strategy where the paramagnetic ions are introduced into a matrix of some biocompatible material. Paramagnetic Gd^3+^ has been incorporated into biocompatible materials such as hydroxyapatite [11], zirconia [12], and carbon [13,14]. Correspondingly, the region of interest on MRI imaging can be brighter (positive agent) because of the longitudinal relaxation that is caused by the presence of gadolinium ions. On the other hand, the negative contrast agents are more effective for bone marrow, liver, and spleen contrast enhancement [2,15]. Negative contrast nanoprobes can be prepared using a biocompatible material that has been doped with dysprosium or holmium ions [4,16]. These nanoprobes can generate strong MR signals thanks to the high magnetic moments of the Dy^3+^ and Ho^3+^ ions and the larger density of the magnetic ions per surface area. In this study, to the best of our knowledge, we present the first report on the synthesis and analysis of carbon dots doped with dysprosium (Dy-CDs) ions. Thus, the main aim of this study is to demonstrate the applicability of the magneto-fluorescent Dy-CDs for simultaneous MR and optical imaging of living cells.

## 2. Experimental

### 2.1. Synthesis Process

Analytical grade dextrose C_6_H_12_O_6_ (≥99.5%) and dysprosium (III) chloride hexahydrate DyCl_3_ × 6H_2_O (99.9%) were purchased from Sigma-Aldrich (St. Louis, MO, USA) and were used as they were received. The carbon nanodots were prepared according to the reported protocols with some modification [17,18]. The solution was prepared by dissolving 2 g of dextrose in 30 mL of deionized (DI) water and was mixed with 0.1 mM of DyCl_3_ × 6H_2_O under continuous stirring. Next, the prepared mixtures were sealed and heated at 190 °C for 2 h. The obtained light-yellow solutions were centrifuged at 12,000 rpm for 30 min. To eliminate the unreacted products, the collected Dy-CDs were purified by dialysis in DI water for 12 h. The recollected Dy-CDs were dispersed in DI water for measurements.

### 2.2. Characterization

The transmission electron microscopy (TEM, JEM-2100, JEOL Ltd., Tokyo, Japan) was utilized to study the morphology of the Dy-CDs. The dynamic light scattering (DLS) size measurements were performed using a Nano ZS Zetasizer (Malvern Instruments Ltd., Malvern, UK). The photoluminescence (PL) excitation and emission spectra of the Dy-CDs were measured using a fluorescence spectrophotometer (Hitachi F-7000, Hitachi Ltd., Tokyo, Japan). The T_2_-weighted images were obtained using a 1.5 T small animal MRI scanner (Siemens Healthinners, Enlargen, Germany). The measurement parameters that were used were as follows: repetition time/echo time (TR/TE) = 2009 ms/9 ms, field of view (FOV) = 160 mm × 160 mm, slice thickness = 5 mm, matrix = 256 × 256, and number of excitations (NEX) = 1. All of the measurements were performed at a room temperature of 22 ± 1 °C.

## 3. Results and Discussion

**Figure 1:**
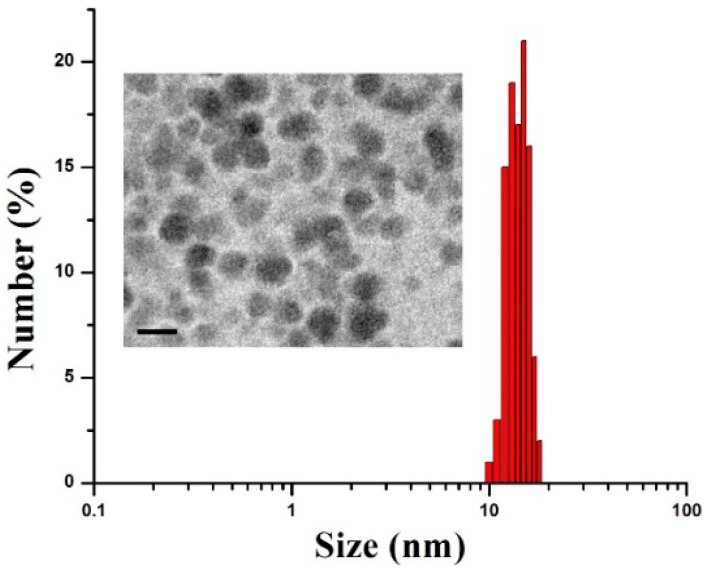
Size-distribution and transmission electron microscopy (TEM) image (scale bar = 20 nm) of prepared carbon dots doped with dysprosium (Dy-CDs).

The dissolved Dy^3+^ ions could be embedded in the carbon nanoprobes to form the paramagnetic Dy-CDs. According to our preliminary results, the size of these nanoprobes could be varied by changing the reaction time, reaction temperature, and dextrose concentration. The reaction time and concentration of the precursors were optimized in this study for the preparation of the fluorescent Dy-CDs with a high transverse relaxivity value. Figure 1 shows the size-distribution and a typical TEM image (inset) of synthesized Dy-CDs. One can see that the prepared Dy-CDs exhibited a nearly spherical shape, with the mean sizes in the range of 12–17 nm. In addition, it should be noted that the prepared Dy-CDs were highly dispersible in an aqueous solution and maintain colloidal stability for several months.

**Figure 2:**
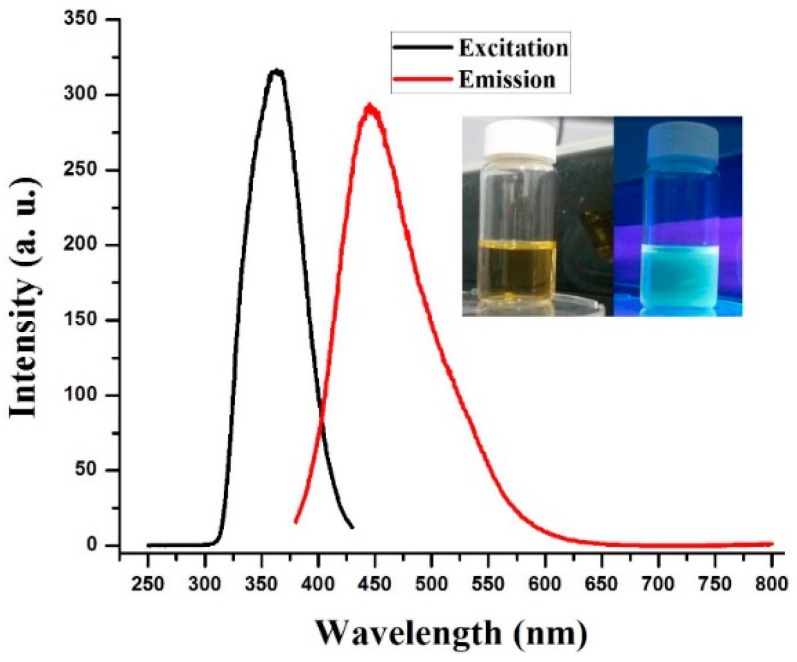
Photoluminescence (PL) excitation and emission spectra of the prepared Dy-CDs. Inset is a digital photo of the Dy-CDs aqueous solution, taken under daylight (left) and 365 nm UV light (right).

The optical properties of the prepared Dy-CDs solution were analyzed using a PL spectroscopy. Figure 2 shows the typical PL excitation and emission spectra of the Dy-CDs solution. One can see that the aqueous solution of the Dy-CDs appeared to be light-brown colored and transparent (Figure 2 inset). The prepared Dy-CDs solution exhibited an excitation-dependent emission, which was a characteristic feature for all of the carbon dots (not shown here). The excitation peak of the Dy-CDs solution reached the maximum value at ~363 nm. Upon a direct 363 nm excitation, the Dy-CDs solution showed the broad blue–green emission peak (Figure 2 inset) with a maximum at 452 nm. It should be mentioned that the excitation and emission peaks of the Dy-CDs solutions were red-shifted compared to the reported studies [13,14]. This red-shift was most likely as a result of the Dy and Cl doping during the synthesis process. The quantum yield (QY) of the prepared Dy-CDs solutions were measured using a quinine bisulfate solution, as the standard was 6.7%. Thus, the prepared Dy-CDs ccould be utilized for the fluorescence imaging of living cells [13,14].

**Figure 3:**
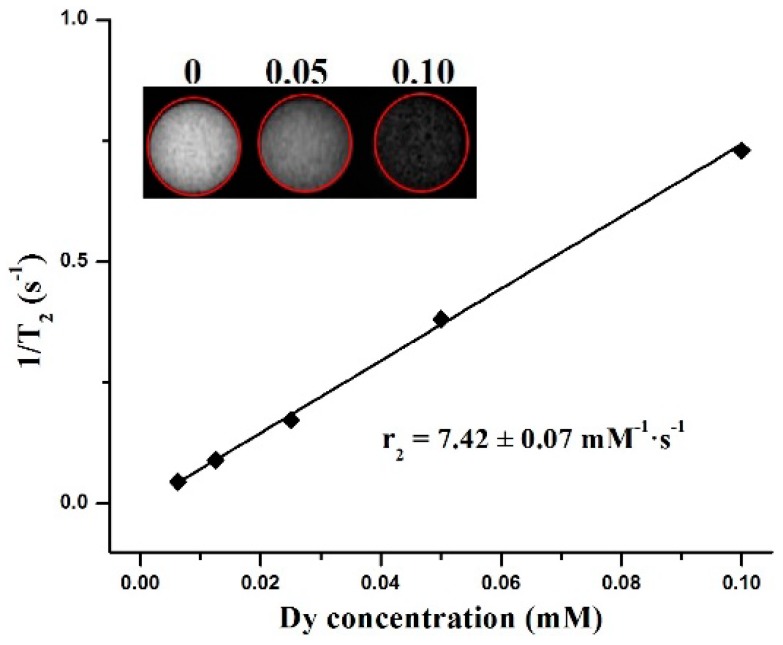
Transverse relaxation rate (1/T_2_) vs. Dy^3+^ concentration (mM). Inset is the transverse relaxation images of Dy-CDs solution at different concentrations.

A small animal MRI scanner was used further in order to confirm the ability of the Dy-CDs that were to be used as contrast agents. It should be noted that the transverse relaxation was not observed in the undoped carbon dots. Figure 3 shows the 1/T_2_ curve that was measured as a function of the total Dy^3+^ concentration. The transverse relaxivity rate (r_2_) was estimated from a linear fit of 1/T_2_ vs. the Dy^3+^ concentration. According to the linear fitting, the r_2_ was calculated to be 7.42 ± 0.07 mM^−1^s^−1^. The obvious darkening of the imaging site (Figure 3 inset) confirmed that the Dy^3+^ ions were successfully introduced into the carbon dots and that the Dy-CDs could be used as a promising negative contrast agent. The Dy^3+^ and Ho^3+^ based contrast agents were especially suitable for high magnetic fields, because the r_2_ value would increase with the increasing the magnetic field [16]. On the other hand, it was necessary to check how the variations of the Dy^3+^ doping concentrations affected the transverse relaxivity rate of the Dy-CDs. In addition, the toxicity of the Dy-CDs should have also been tested using several cell lines, despite the fact that the Gd-doped CDs exhibited a low toxicity [13,14]. We have planned to address the above-mentioned issues in our next full study.

## 4. Conclusions

In summary, the bimodal Dy-doped CDs contrast nanoprobes were prepared using a facile hydrothermal synthesis method. The TEM and DLS measurements showed that the average size of the prepared Dy-CDs was about 12–17 nm. According to the PL study, the prepared Dy-CDs emitted a blue–green emission with a maximum at 452 nm. The MRI measurements of the Dy-CDs revealed that the transverse relaxivity rate (r_2_) value of the sample was 7.42 ± 0.07 mM^−1^s^−1^. We strongly believe that the prepared bifunctional Dy-CDs can be of great interest for MRI and fluorescence imaging of living cells.
